# Current status and factors influencing oral anticoagulant therapy among patients with non-valvular atrial fibrillation in Jiangsu province, China: a multi-center, cross-sectional study

**DOI:** 10.1186/s12872-020-01330-6

**Published:** 2020-01-16

**Authors:** Ting Liu, Hui-li Yang, Lan Gu, Jie Hui, Ojo Omorogieva, Meng-xiao Ren, Xiao-hua Wang

**Affiliations:** 1 Department of Cardiology, The First Affiliated Hospital of Soochow University, Suzhou, 215006 People’s Republic of China; 2 School of Nursing, Medical College of Soochow University, Suzhou, 215006 People’s Republic of China; 3 Boxi Medical Center, The First Affiliated Hospital of Soochow University, Suzhou, 215006 People’s Republic of China; 4 Department of Adult Nursing and Paramedic Science, University of Greenwich, SE92UG, London, UK

**Keywords:** Atrial fibrillation, Anticoagulant therapy, CHA_2_DS_2_-VASc scores, Influencing factors, China

## Abstract

**Background:**

It has been reported that oral anticoagulation (OAC) is underused among Chinese patients with non-valvular atrial fibrillation (NVAF). Non-vitamin K antagonist oral anticoagulants (NOAC) have been recommended by recent guidelines and have been covered since 2017 by the Chinese medical insurance; thus, the overall situation of anticoagulant therapy may change. The aim of this study was to explore the current status of anticoagulant therapy among Chinese patients with NVAF in Jiangsu province.

**Methods:**

This was a multi-center, cross-sectional study that was conducted in seven hospitals from January to September in 2017. The demographic characteristics and medical history of the patients were collected by questionnaire and from the medical records. Multivariate logistic regression was used to identify factors associated with anticoagulant therapy.

**Results:**

A total of 593 patients were included in the analysis. A total of 35.6% of the participants received OAC (11.1% NOAC and 24.5% warfarin). Of those patients with a high risk of stroke, 11.1% were on NOAC, 24.8% on warfarin, 30.6% on aspirin, and 33.6% were not on medication. Self-paying, duration of AF ≥5 years were negatively associated with anticoagulant therapy in all patients (OR 1.724, 95% CI 1.086~2.794; OR 1.471, 95% CI 1.006~2.149, respectively), whereas, permanent AF was positively associated with anticoagulant therapy (OR 0.424, 95% CI 0.215~0.839). Among patients with high risk of stroke, self-paying and increasing age were negatively associated with anticoagulant therapy (OR 2.305, 95% CI 1.186~4.478; OR 1.087, 95% CI 1.041~1.135, respectively).

**Conclusions:**

Anticoagulant therapy is positively associated with permanent AF and negatively associated with self-paying, duration of AF > 5 years. Furthermore, the current status of anticoagulant therapy among Chinese patients with NVAF in Jiangsu province does not appear optimistic. Therefore, further studies should focus on how to improve the rate of OAC use among NVAF patients. In addition, policy makers should pay attention to the economic situation of the patients with NVAF using NOAC.

**Trial registration:**

2,017,029. Registered 20 March 2017 (retrospectively registered).

## Background

Atrial fibrillation (AF) is the most common cardiac arrhythmia in clinical practice. It has been reported that the prevalence of AF in the elderly Chinese population was 1.8% [1]. With an increasingly aging population and the incidence of AF-related risk factors, this number is expected to rise. It was predicted that there would be 5.2 million men and 3.1 million women aged > 60 years with AF in China by 2050 [2], which will be a major social health problem. AF is an independent risk factor for stroke, and AF-related stroke accounts for 20% of all strokes [3, 4]. Apart from this, AF is independently associated with a 2-fold increased risk of all-cause mortality in women and a 1.5-fold increase in men [5–7], imposing a considerable medical burden on individuals and health care systems.

A cornerstone of the management AF is to prevent AF-related stroke and systemic embolism. Recently, some major guidelines have recommended oral anticoagulation (OAC) for patients with non-valvular atrial fibrillation (NVAF), especially those at moderate to high risk of thromboembolism based on CHA_2_DS_2−_VAS_C_ scores, for reducing risk of stroke [8, 9]. Currently, OAC used in clinical practice include vitamin K antagonists (VKAs, e.g., warfarin) and non-vitamin K antagonist oral anticoagulants (NOAC, e.g., dabigatran, rivaroxaban, apixaban, and edoxaban).

However, as far as we know, the use of OAC is suboptimal in China. In 2016, a prospective, multi-center study conducted in China showed that 36.5% of NVAF patients with CHA_2_DS_2_-VASc scores > 2 received OAC (warfarin and NOAC) [10]. This study also reported that a high proportion of AF patients (> 41%) used aspirin as an anticoagulant [10]. In 2017, Yang surveyed the use of OAC in AF patients in one hospital and concluded that 35.0% of patients received OAC, and 11.0% received NOAC [11]. The reason for the low OAC usage may be due to the fact that, although warfarin is widely used among AF patients due to its effectiveness and low price, it has a narrow therapeutic interval and needs frequent monitoring and dose adjustments [9]. Additionally, some NVAF patients with cardiovascular diseases chose antiplatelet drugs (e.g., aspirin) to prevent embolism, which may influence the use of OAC. Compared to VKAs, NOAC was confirmed safer, slightly more effective and more convenient [12–14]. However, studies have found that high price is one of the main reasons that limit patients’ use of NOAC [10, 11]. In order to reduce the financial burden of patients, the Chinese medical insurance has covered NOAC since 2017. As a result, patients are paying less for NOAC. The current anticoagulant therapy status among NVAF patients is unknown in Jiangsu province, which is an economically developed and densely populated area in China.

## Methods

### The aim, design and setting of the study

The aim of this multi-center, cross-sectional study was to understand the current status of anticoagulant therapy among Chinese patients with NVAF in Jiangsu province. As this is an investigative study and based on the reported rate of the OAC use which is put at 35% [10], the sample size of the number of people to be surveyed was estimated to be 972. In addition, taking into account 20% of the invalid questionnaire, 1167 patients should be surveyed.

Patients were enrolled from the departments of cardiology of seven hospitals in the different areas of Jiangsu province from January to September in 2017. Three of the hospitals were general tertiary hospitals and four were local hospitals. Eligible patients were diagnosed as AF by electrocardiogram or ambulatory electrocardiogram recorder according to the guideline of the European Society of Cardiology (ESC) [9] and were ≥ 18 years. Exclusion criteria were as follows: (1) patients were with valvular disease or mechanical valve; (2) rheumatic heart disease; (3) reversible AF (caused by hyperthyroidism, acute myocardial infarction and so on); (4) malignant tumors and blood diseases; (5) underwent surgeries within three months.

### Data collection

The patients’ sociodemographic characteristics (including sex, age, payment, educational level), information on AF (including type and duration of AF), medical history (including hypertension, diabetes mellitus, hyperlipidemia, coronary heart disease, peripheral artery disease, stroke, transient ischemic attack, myocardial infarction, gastrointestinal bleeding, cerebral hemorrhage, impaired renal/liver function) and use of any oral anticoagulants were obtained by patient-reported questionnaire and medical records. Investigators accepted uniform training, guided patients to fill out the questionnaire and obtained their medical history from medical records. Following that, the CHA_2_DS_2_-VASc scores and HAS-BLED scores were calculated for each patient based on their data.

The diagnosis of diabetes and hypertension were reported by patients or collected from the medical records. Diabetes was diagnosed according to the 1999 World Health Organization criteria for diabetes [15]. It was defined based on fasting capillary whole blood glucose level (≥ 6.1 mmol/L) or plasma glucose measurement (FPG ≥ 7.0 mmol/L and/or 2-h postprandial blood glucose ≥11.1 mmol/L during an oral glucose tolerance test). Hypertension was defined according to 2010 Chinese guidelines for the management of hypertension [16], which was described as systolic blood pressure (SBP) ≥ 140 mmHg, diastolic blood pressure (DBP) ≥ 90 mmHg, and use of antihypertensive medicine within two weeks.

### Risk stratification of stroke and bleeding

The CHA_2_DS_2_-VASc score was calculated for each patient to categorize the risk of stroke by assigning 1 point each for age between 65 and 74 years, a history of hypertension, diabetes mellitus, congestive heart failure, vascular disease (CAD or peripheral artery disease), female sex and 2 points each for a history of stroke/TIA/thromboembolism and age ≥ 75 years [9]. The total possible score was 9 points and higher scores indicated a higher risk of stroke. According to the 2016 ESC guideline, the high risk of stroke is CHA_2_DS_2_VASc ≥ 2 for males and CHA_2_DS_2_VASc ≥ 3 for females [9].

The HAS-BLED scores were also calculated for each patient to categorize the risk of bleeding by assigning 1 point each for hypertension, abnormal renal/liver function, stroke, bleeding history or predisposition, labile INR, elderly (age > 65 years), drugs/alcohol concomitantly [9]. The total possible score on this scale was also 9 points and score ≥ 3 indicated a high risk of bleeding [9].

### Outcome variable

The outcome variable was defined as whether patients received OAC (NOAC or warfarin) or not (aspirin or no medication), and it was coded as “1 = Yes” and “2 = No” for multivariate logistic model.

### Statistical analysis

Patients were divided into two groups according to the use of anticoagulant: OAC (NOAC or warfarin) or no OAC (aspirin or no medication). Categorical variables, expressed as numbers and percentages, were compared by Wilcoxon rank-sum test, Chi-square test. Univariate analyses were used to screen significant variables. Variables with *p* values < 0.1 and variables that may be associated with anticoagulant therapy were included in the multivariate analyses. The factors that make up CHA_2_DS_2_-VASc score were adjusted as the confounding variables in multivariate models. Subgroup analysis was performed in patients with a history of stroke. Statistical significance was considered at *p* <  0.05. All analyses were performed using SPSS 20.0 software.

## Results

**Fig. 1:**
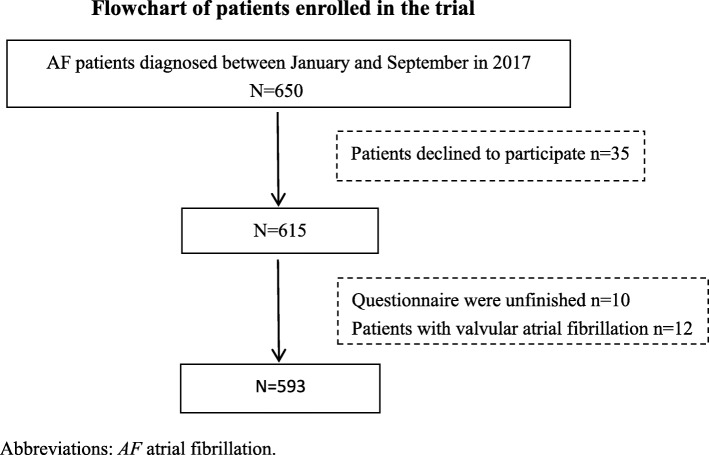
Flowchart of patients enrolled in the trial, Abbreviations: *AF* atrial fibrillation

A total of 650 patients were recruited from seven hospitals in the study, while data from 593 patients (91.2%) were analyzed (Fig. 1). Among eligible patients, 47.4% were female and 21.9% had no medical insurance. The majority of patients (87.2%) were older than 60 years and more than half of the patients had a primary school education or below. 56.3% of the patients had a duration of AF which was less than 5 years; 72.8% of patients had a high risk of stroke; 10.5% had a high risk of bleeding. The prevalence of prior stroke was 11.1%. Detailed demographics and medical history are shown in Table 1.

**Table 1:** Demographic and clinical characteristics in patients with NVAF *n* = 593

Variables	Classification	N (%)
Gender	Female	281 (47.4)
Payment	Self-paying	130 (21.9)
Age, years	< 60	76 (12.8)
	60~69	157 (26.5)
	70~79	205 (34.6)
	≥80	155 (26.1)
Education	Primary school or below	316 (53.3)
	Junior high school	145 (24.5)
	Senior high school and above	132 (22.2)
AF type	Paroxysmal	381 (64.3)
	Persistent	172 (29.0)
	Permanent	40 (6.7)
Duration, years	< 5	335 (56.3)
Severity of symptom	Asymptomatic	61 (10.3)
	Mild	334 (56.3)
	Moderate	169 (28.5)
	Severe	29 (4.9)
Stroke	Yes	66 (11.1)
Concomitant disease		
Hypertension	Yes	359 (60.5)
Diabetes mellitus	Yes	118 (19.9)
Coronary heart disease	Yes	138 (23.2)
Heart failure	Yes	94 (15.8)
Vascular disease	Yes	27 (4.6)
Hyperlipidemia	Yes	38 (6.4)
Impaired renal/liver function	Yes	29 (4.9)
HAS-BLED, score	< 3	531 (89.5)
CHA_2_DS_2_-VAS_C_, score	Male: 0~2; female: 0~1	161 (27.2)
	Male ≥3; female ≥2	432 (72.8)

Abbreviations: *AF* Atrial fibrillation, *CHA*_*2*_*DS*_*2*_*-VASc* Congestive heart failure, hypertension, age ≥ 75 years, diabetes mellitus, prior stroke, transient ischemic attack, or thromboembolism, vascular disease, age 65–74 years,sex category (female), *HAS-BLED* Hypertension, abnormal renal and/or liver function, stroke, bleeding history or predisposition, labile international normalized ratio, elderly (age > 65 years), drugs and/or alcohol concomitantly, *NVAF* Non-valvular atrial fibrillation, *TIA* Transient ischemic attack

Among patients with a high risk of stroke, 11.1% were on NOAC, 24.8% on warfarin and 30.6% on aspirin, while 33.6% were not on medication. Of the patients with low risk of bleeding, 10.5% were on NOAC, 24.7% on warfarin, 28.6% on aspirin and 36.2% were not on medication. For the patients whose HAS-BLED score was ≥3, the corresponding numbers with respect to taking NOAC, warfarin, aspirin and no medication were 16.1, 22.6, 27.4 and 33.9%, respectively. The status of anticoagulant therapy based on the classification of CHA_2_DS_2_-VASc score and HAS-BLED score are shown in Table 2.

**Table 2:** Status of anticoagulant therapy among patients with NVAF at the different risk levels of stroke and bleeding

	NOAC, n(%)	Warfarin, n(%)	Aspirin, n(%)	No medication, n(%)
CHA_2_DS_2_-VASc				
Male: 0~2; female: 0~1	18 (11.2)	38 (23.6)	37 (23.0)	68 (42.2)
Male ≥3; female ≥2	48 (11.1)	107 (24.8)	132 (30.5)	145 (33.6)
HAS-BLED				
< 3	56 (10.5)	131 (24.7)	152 (28.6)	192 (36.2)
≥ 3	10 (16.1)	14 (22.6)	17 (27.4)	21 (33.9)

Abbreviations: *CHA*_*2*_*DS*_*2*_*-VASc* Congestive heart failure, hypertension, age ≥ 75 years, diabetes mellitus, prior stroke, transient ischemic attack, or thromboembolism, vascular disease, age 65–74 years, sex category (female), *HAS-BLED* Hypertension, abnormal renal and/or liver function, stroke, bleeding history or predisposition, labile international normalized ratio, elderly (age > 65 years), drugs and/or alcohol concomitantly, *NOAC* Non-vitamin K antagonist oral anticoagulant, *NVAF* Non-valvular atrial fibrillation

The results of the univariate analysis showed that variables including payment, age, duration of AF tended to be related with anticoagulant therapy in all patients, and payment, age, duration of AF and hypertension tended to be related with anticoagulant therapy in patients with a high risk of stroke. (Table 3).

**Table 3:** Relationship between demographic, clinical characteristics and anticoagulant therapy in all patients and those with high risk of stroke

Variables	Classification	All patients n(%)		z/χ^2^	*p* value	patients with high risk of stroke n(%)		z/χ^2^	*p* value
		OAC	no OAC			OAC	no OAC		
Gender	Male	114 (37.5)	198 (63.5)	1.795	0.180 ^α^	78 (35.8)	140 (64.2)	0.602	0.438 ^α^
	Female	88 (31.3)	193 (68.7)			69 (32.2)	145 (67.8)		
Payment	Self-paying	32 (24.6)	98 (75.4)	6.618	0.010 ^α^	15 (21.1)	56 (78.9)	6.299	0.012 ^α^
	Medical insurance	170 (36.7)	293 (63.3)			132 (36.6)	229 (63.4)		
Age	< 60	31 (40.8)	45 (59.2)	−3.017	0.003 ^β^	11 (73.3)	4 (26.7)	24.304	0.000 ^β^
	60~69	57 (36.3)	100 (63.7)			35 (39.3)	54 (60.7)		
	70~79	81 (39.5)	124 (60.5)			68 (39.1)	106 (60.9)		
	≥80	33 (21.3)	122 (78.7)			33 (21.4)	121 (78.6)		
Education	Primary school or below	103 (32.6)	213 (67.4)	−0.290	0.771 ^β^	84 (32.7)	173 (67.3)	1.402	0.496 ^β^
	Junior high school	58 (40.0)	87 (60.0)			35 (39.3)	54 (60.7)		
	Senior high school and above	41 (31.1)	91 (68.9)			28 (32.6)	58 (67.4)		
AF type	Paroxysmal	122 (32.0)	259 (68.0)	−1.697	0.090 ^β^	82 (31.8)	176 (68.2)	2.298	0.317 ^β^
	Persistent	60 (34.9)	112 (65.1)			50 (35.7)	90 (64.3)		
	Permanent	20 (50.0)	20 (50.0)			16 (45.7)	19 (54.3)		
Duration	< 5 years	129 (38.5)	206 (61.5)	6.768	0.009 ^α^	89 (38.2)	144 (61.8)	4.774	0.029 ^α^
	≥5 years	73 (28.3)	185 (71.7)			58 (29.1)	141 (70.9)		
Symptom of severity	Asymptomatic	28 (45.9)	33 (54.1)	−0.951	0.342 ^β^	20 (46.5)	23 (53.5)	4.143	0.246 ^β^
	Mild	108 (32.3)	226 (67.7)			77 (32.5)	160 (67.5)		
	Moderate	55 (32.5)	114 (67.5)			42 (31.6)	91 (68.4)		
	Severe	11 (37.9)	18 (62.1)			8 (42.1)	11 (57.9)		
Hypertension	Yes	129 (35.9)	230 (64.1)	1.686	0.194 ^α^	115 (37.5)	192 (62.5)	5.565	0.018 ^α^
	No	72 (30.8)	162 (69.2)			32 (25.6)	93 (74.4)		
Vascular disease	Yes	7 (25.9)	20 (74.1)	0.802	0.371 ^α^	18 (28.1)	46 (71.9)	1.166	0.280 ^α^
	No	194 (34.3)	372 (65.7)			129 (35.1)	239 (64.9)		
Diabetes mellitus	Yes	46 (39.0)	72 (61.0)	1.702	0.192 ^α^	46 (40.4)	68 (59.6)	2.758	0.097 ^α^
	No	155 (32.6)	320 (67.4)			101 (31.8)	217 (68.2)		
Coronary heart disease	Yes	41 (29.7)	97 (70.3)	1.406	0.236 ^α^	36 (31.6)	78 (68.4)	0.414	0.520 ^α^
	No	160 (35.2)	295 (64.8)			111 (34.9)	207 (65.1)		
Heart failure	Yes	36 (37.9)	59 (62.1)	0.537	0.464 ^α^	30 (37.0)	51 (63.0)	0.402	0.526 ^α^
	No	155 (32.6)	320 (67.4)			117 (33.3)	234 (66.7)		
Hyperlipidemia	Yes	8 (21.1)	30 (78.9)	2.989	0.084 ^α^	5 (20.0)	20 (80.0)	2.326	0.127 ^α^
	No	193 (34.8)	362 (65.2)			142 (34.9)	265 (65.1)		
Stroke	Yes	19 (28.8)	47 (71.2)	0.865	0.352 ^α^	19 (29.2)	46 (70.8)	0.588	0.443 ^α^
	No	182 (34.5)	345 (65.5)			128 (34.9)	239 (65.1)		
Impaired renal/liver function	Yes	13 (44.8)	16 (55.2)	1.626	0.202 ^α^	12 (44.4)	15 (55.6)	1.392	0.238 ^α^
	No	188 (33.3)	376 (66.7)			135 (33.3)	270 (66.7)		
HAS-BLED, score	< 3	180 (33.9)	351 (66.1)	0.062	0.803 ^α^	125 (33.8)	245 (66.2)	0.068	0.794 ^α^
	≥3	22 (35.5)	40 (64.5)			22 (35.5)	40 (64.5)		
CHA_2_DS_2_-VASc, score	Male: 0~2; female: 0~1	56 (34.8)	105 (65.2)	0.062	0.804 ^α^				
	Male ≥3; female ≥2	155 (35.9)	277 (64.1)						

Abbreviations: *AF* Atrial fibrillation, *CHA*_*2*_*DS*_*2*_*-VASc* Congestive heart failure, hypertension, age ≥ 75 years,diabetes mellitus, prior stroke, transient ischemic attack, or thromboembolism, vascular disease, age 65–74 years, sex category (female), *HAS-BLED* Hypertension, abnormal renal and/or liver function, stroke, bleeding history or predisposition, labile international normalized ratio, elderly (age > 65 years), drugs and/or alcohol concomitantly, *NOAC* Non-vitamin K antagonist oral anticoagulant

α: χ^2^-test; β: Wilcoxon rank-sum test

Multivariate logistic regression analysis indicated that self-paying, increasing age, duration of AF ≥5 years were negatively associated with anticoagulant therapy in all patients, whereas permanent AF was positively associated with anticoagulant therapy. Self-paying was the strongest predictor of no anticoagulant therapy [odds ratio (OR) 1.824, 95% confidence interval (CI) 1.136–2.928], followed by the duration of AF ≥5 years (OR 1.505, 95% CI 1.035~2.189) and increasing age (OR 1.030, 95% CI 1.010~1.050). Patients with permanent AF were more likely to receive anticoagulant therapy (OR 0.406, 95% CI 0.206~0.800). In the adjusted model, increasing age was not significantly associated with anticoagulant therapy. However, self-paying (OR 2.234, 95% CI 1.167~4.277) and increasing age (OR 1.055, 95% CI 1.028~1.082) were associated with no anticoagulant therapy in patients with a high risk of stroke, and these factors were still significant in the adjusted model. (Tables 4 & 5).

**Table 4:** Factors associated with anticoagulant therapy in all patients and those with high risk of stroke (Multivariate logistic regression)

	All patients			patients with high risk of stroke		
Variables	B	OR (95% *CI*)	*P* value	B	OR (95% *CI*)	*P* value
Gender, Female	0.210	1.234 (0.848~1.796)	0.272	0.043	1.044 (0.681~1.601)	0.844
Payment, Self-paying	0.601	1.824 (1.136~2.928)	0.013	0.884	2.234 (1.167~4.277)	0.015
Age (per 1 year increase)	0.029	1.030 (1.010~1.050)	0.003	0.053	1.055 (1.028~1.082)	< 0.001
Education	−0.180	0.835 (0.553~1.260)	0.391	−0.060	0.941 (0.565~1.568)	0.817
AF type, Permanent	−0.902	0.406 (0.206~0.800)	0.009	−0.615	0.541 (0.253~1.157)	0.113
Duration, ≥5 years	0.409	1.505 (1.035~2.189)	0.033	0.286	1.332 (0.863~2.055)	0.196
Severity of symptom	−0.003	0.997 (0.774~1.283)	0.979	0.057	1.059 (0.598~1.874)	0.845
HAS-BLED, score ≥ 3	−0.316	0.729 (0.384~1.385)	0.334	−0.196	0.822 (0.446~1.516)	0.530
CHA_2_DS_2_-VAS_C,_ (per 1score increase)	−0.117	0.890 (0.747~1.059)	0.190	−0.058	0.944 (0.736~1.210)	0.647
Hypertension				0.456	1.578 (0.966~2.578)	0.068

Abbreviations: *AF* Atrial fibrillation, *CHA*_*2*_*DS*_*2*_*-VASc* Congestive heart failure, hypertension, age ≥ 75 years, diabetes mellitus, prior stroke, transient ischemic attack, or thromboembolism, vascular disease, age 65–74 years, sex category (female), *CI* Confidence interval, *HAS-BLED* Hypertension, abnormal renal and/or liver function, stroke, bleeding history or predisposition, labile international normalized ratio, elderly (age > 65 years), drugs and/or alcohol concomitantly, *OR* Odds ratio

**Table 5:** Factors associated with anticoagulant therapy in all patients and those with high risk of stroke (Multivariate logistic regression)

	All patients			Patients with high risk of stroke		
Variables	B	OR (95% *CI*)	*P* value	B	OR (95% *CI*)	*P* value
Gender, Female	0.185	1.203 (0.820~1.763)	0.345	0.091	1.096 (0.641~1.874)	0.739
Payment, Self-paying	0.555	1.742 (1.086~2.794)	0.021	0.835	2.305 (1.186~4.478)	0.014
Age (per 1 year increase)	0.024	1.024 (0.997~1.053)	0.085	0.084	1.087 (1.041~1.135)	< 0.001
Education	−0.108	0.897 (0.590~1.364)	0.612	−0.069	0.934 (0.555~1.572)	0.796
AF type, Permanent	−0.857	0.424 (0.215~0.839)	0.014	−0.552	0.576 (0.264~1.255)	0.165
Duration, ≥5 years	0.386	1.471 (1.006~2.149)	0.046	0.286	1.332 (0.858~2.067)	0.202
Severity of symptom	0.132	1.141 (0.715~1.819)	0.580	0.043	1.044 (0.586~1.860)	0.884
HAS-BLED, score ≥ 3	−0.339	0.712 (0.369~1.375)	0.312	−0.289	0.749 (0.364~1.540)	0.432
CHA_2_DS_2_-VAS_C_,(per1score increase)	−0.108	0.897 (0.713~1.130)	0.357	0.015	1.015 (0.750~1.374)	0.923
Hypertension				0.465	1.592 (0.933~2.717)	0.088

Adjustment variable: congestive heart failure, hypertension, age ≥ 75 years, diabetes mellitus, prior stroke, transient ischemic attack, orth romboembolism, vascular disease, sex category (female)

Abbreviations: *AF* atrial fibrillation, *CHA*_*2*_*DS*_*2*_*-VASc* Congestive heart failure, hypertension, age ≥ 75 years, diabetes mellitus, prior stroke, transient ischemic attack, or thromboembolism, vascular disease, age 65–74 years, sex category (female), *CI* Confidence interval, *HAS-BLED* Hypertension, abnormal renal and/or liver function, stroke, bleeding history or predisposition, labile international normalized ratio, elderly (age > 65 years), drugs and/or alcohol concomitantly, *OR* Odds ratio

In subgroup analysis, increasing age was negatively associated with anticoagulant therapy among patients with prior stroke (OR 1.075, 95% CI 1.016~1.139). Furthermore, self-paying was not only the influencing factor of warfarin use, but also the influencing factor of NOAC use (OR 1.816, 95% CI 1.089~3.028; OR 2.311, 95% CI 1.556~3.433, respectively). Details are shown in Tables 6 and 7.

**Table 6:** Factors associated with anticoagulant therapy in patients with prior stroke

	B	OR (95% *CI*)	*p* value
Univariate analysis			
Gender, female	0.319	1.376 (0.473~4.004)	0.559
Self-paying	0.360	1.434 (0.401~5.130)	0.580
Age, (per 1 year increase)	0.055	1.057 (1.005~1.112)	0.031
≥ 75 years old	1.107	3.025 (1.001~9.142)	0.050
Education	− 0.279	0.757 (0.220~2.608)	0.659
AF type, Permanent	−0.235	0.791 (0.132~4.726)	0.797
Duration, ≥5 years	−0.276	0.759 (0.259~2.224)	0.615
Severity of symptom	0.882	2.415 (0.499~11.682)	0.273
HAS-BLED, score ≥ 3	−0.706	0.494 (0.167~1.455)	0.200
CHA_2_DS_2_-VASc, (per 1 score increase)	0.228	1.257 (0.851~1.856)	0.251
Vascular disease	− 0553	0.5752 (0.177~1.870)	0.358
Congestive heart failure	0.205	1.227 (0.120~12.597)	0.863
Diabetes mellitus	0.535	1.708 (0.518~5.631)	0.379
Hypertension	−0.343	0.710 (0.238~2.116)	0.538
Multivariate analysis			
Age, (per 1 year increase)	0.073	1.075 (1.016~1.139)	0.013
≥ 75 years old	0.459	1.583 (0.239~10.483)	0.634
Self-paying	0.342	1.407 (0.359~5.515)	0.624
AF type, Permanent	−0.568	0.567 (0.079~4.066)	0.572
Duration, ≥5 years	−0.302	0.739 (0.220~2.481)	0.625
HAS-BLED, score ≥ 3	−1.061	0.346 (0.102~1.175)	0.089

Abbreviations: *AF* atrial fibrillation, *CHA*_*2*_*DS*_*2*_*-VASc* Congestive heart failure, hypertension, age ≥ 75 years, diabetes mellitus, prior stroke, transient ischemic attack, or thromboembolism, vascular disease, age 65–74 years, sex category (female), *CI* Confidence interval, *HAS-BLED* Hypertension, abnormal renal and/or liver function, stroke, bleeding history or predisposition, labile international normalized ratio, elderly (age > 65 years), drugs and/or alcohol concomitantly, *OR* Odds ratio

**Table 7:** The relationship between self-paying and warfarin or NOAC use (Univariate analysis)

	B	OR (95% CI)	*p* value
Self-paying (warfarin)	0.597	1.816 (1.089~3.028)	0.022
Self-paying (NOAC)	0.838	2.311 (1.556~3.433)	< 0.001

Abbreviations: *NOAC* Non-vitamin K antagonist oral anticoagulant, *CI* Confidence interval, *OR* Odds ratio

## Discussion

Anticoagulant therapy is the cornerstone of AF management. The ESC guideline recommended OAC for patients with AF who are at high risk of stroke to prevent stroke [9]. We investigated the OAC usage in a real-world cohort among Chinese patients with NVAF in Jiangsu province and found that the overall situation of OAC usage does not appear optimistic, even in NVAF patients at high risk of stroke. In our study, only 35.6% of NVAF patients with different risks received OAC which is lower than the data shown by the EURObservational Research Programme on Atrial Fibrillation Pilot Survey, which found that 80.5% of AF patients with CHA_2_DS_2_VASc scores ≥1 received OAC [17]. The proportion of NVAF patients receiving warfarin and NOAC were 24.5 and 11.1%, respectively, which are both lower than the data presented by ORBIT-AF study (71% warfarin) [18] and the ORBIT-AF II study (71% NOAC) [19].

The 2016 ESC guideline states that aspirin is not recommended for antithrombotic therapy regardless of the risk of stroke in patients with AF [9]. Although the rate of aspirin usage in this study was lower than it in another study (> 41%) [10], a considerable proportion of patients (28.5%) still used aspirin as an anticoagulant. In addition, 30.5% of patients with high risk of stroke were on aspirin alone, which was much higher than the data of the ORBIT-AF II study (8%) [19]. This might be because the patients enrolled in the study were mainly elderly patients (about 60% of them were ≥ 70 years old) who were commonly using aspirin for prevention of thrombosis. Besides, patients with coronary heart disease who usually take aspirin for treatment, and in this study 23.2% of NVAF patients had coronary heart disease. A recent study in Thailand concluded that the administration of aspirin was one of the reasons for not prescribing anticoagulants in the older population [20]. In addition, more than half of the participating hospitals were local hospitals and there may be gaps in medical resources between hospital at different levels. For example, in primary hospitals, doctors may lack knowledge and understanding of new treatment regimens. However, doctors have the responsibility to tell patients the importance of anticoagulant therapy. In this regard, doctors in primary hospitals should update their knowledge timely in order to educate NVAF patients.

Another finding of the study is that the bleeding risk did not influence the OAC use which is in line with ESC guideline which indicates that a high level of HAS-BLED score is not an excuse to withhold OAC [9]. However, 33.6% of patients with high risk of stroke did not take any anticoagulants in this study, which is consistent with Yang’s study in 2017 [11]. Namely, the high CHA_2_DS_2_-VASC score was not associated with anticoagulant therapy, which reminds us to focus on the phenomenon that patients were not prescribed with OAC suitably according to their risk stratification of stroke, and methods of improving this condition including educational programme should be put in place.

The multivariate logistic analysis showed that in the adjustment model, self-paying, the duration of AF > 5 years was negatively associated with anticoagulant therapy, whereas permanent AF was positively associated with anticoagulant therapy in all patients. Among patients with a high risk of stroke, self-paying and increasing age were negatively associated with anticoagulant therapy.

### Age and payment

The relationship between old age and medication adherence has been as an issue in different studies [10, 21–23]. A population-based retrospective cohort study in Spain demonstrated that younger patients showed poorer primary adherence [22]. However, some studies concluded that old patients had poorer medication adherence [10, 23], which is consistent with the results of this study. One possible explanation is that older people with AF commonly have other comorbidities. About 70% of patients in this study had comorbidity, and of these, 31.4% of patients had two or more comorbidities, which usually leads to polypharmacy, a condition known to influence anticoagulant therapy [24]. In addition, physicians are inclined to overestimate the risk of bleeding when an older patient has both a high risk of bleeding and stroke [25, 26]. In the subgroup analysis, increasing age was also negatively associated with anticoagulant therapy among the patients with prior stroke. Palareti et al. found that previous stroke history was an independent risk factor for oral anticoagulants and blood events [27]. Moreover, patients with stroke history usually have sequela which may limit them to visit doctors, further influencing the OAC use.

Self-paying is a major factor associated with OAC use in the study, especially with NOAC, which was also found in another study [28]. Warfarin needs frequent monitoring for INR, dose adjustment, drug or food interactions and fares to the hospital [9], which limits patients use it. Currently in China, the medical insurance could reimburse the cost of NOAC for inpatients. For outpatients, 70% of cost could be reimbursed, and the remaining 30% need to be paid at their own expense. However, most of NVAF patients are outpatients, which means that patients payments for NOAC remain high (about $1740/y). Therefore, the economic burden can explain the low OAC usage to some extent. In addition, as older people usually have lower incomes, the old age of the patients may strengthen the connection between self-paying and no anticoagulant therapy. Thus, the authorities providing insurance policy should pay attention to this health economics issue to help low income patients to improve OAC usage.

### Duration and type of AF

Previous studies have shown that the rate of OAC use has decreased over time [29]. Besides, the unsatisfactory therapeutic effect also appears to influence adherence to long-term medication use. In this study, compared to those with the duration of AF < 5 years, patients who had the duration of AF ≥ 5 years were less likely to receive anticoagulant therapy. Health education interventions, such as regular educational programme and mobile health technologies, can significantly improve OAC use and drug adherence in patients with NVAF [30, 31]. Therefore, it is necessary to timely evaluate changes in patients’ medication adherence and regularly provide health education for patients with NVAF.

Patients with permanent AF were more likely to receive anticoagulant therapy in this study, which is in line with the study in 2016 by Wang et al. [32]. In 2012, Chiang et al. reported that stroke rates were higher in patients with persistent than paroxysmal AF, while these were highest in patients with permanent AF [33]. The guideline on AF management recommended OAC for stroke prevention, irrespective of the type of AF [8]. Aronis et al. in 2016 [34] and Rizos et al. [35] in 2011 reported that a significant proportion of episodes of stroke in patients with AF occur in patients with a history of paroxysmal AF, varying from 31 to 53%. In view of this, physicians should pay more attention to patients with paroxysmal or persistent AF.

Some limitations of this study must be considered. Most of the data in the study was collected using the self-reported questionnaire, in which the existence of recall bias should be recognized, although this method is a good way to investigate the use of anticoagulants among a large number of NVAF patients. As a multi-center and cross-sectional study, the sample size was small, and the participants were only enrolled from seven hospitals in Jiangsu province. Furthermore, psychosocial factors influencing the OAC use were not considered which may limit the interpretation of the results.

## Conclusions

The results of this study showed that OAC was underused among Chinese NVAF patients in Jiangsu province, especially in patients at high risk of stroke. Anticoagulant therapy is positively associated with permanent AF and negatively associated with self-paying, duration of AF > 5 years. In order to improve the use of OAC, the importance of anticoagulant therapy should be emphasised, and the economic situation of NVAF patients in relation to anticoagulant therapy should be considered.

## Data Availability

The datasets used and analyzed during the current study are available from the corresponding author on reasonable request.

## Abbreviations

AF: Atrial fibrillation
CHA_2_DS_2_-VASc: Congestive heart failure, hypertension, age ≥ 75 (doubled), diabetes, stroke (doubled), vascular disease, age 65–74, and sex (female)
CI: Confidence interval
ESC: European society of cardiology
HAS-BLED: Hypertension, abnormal renal/liver function (1 point each), stroke, bleeding history or predisposition, labile international normalized ratio, elderly (> 65 years), drugs/alcohol concomitantly (1 point each)
NOAC: Non-vitamin K antagonist oral anticoagulant
NVAF: Non-valvular atrial fibrillation
OAC: Oral anticoagulation
OR: Odds ratio
TIA: Transient ischemic attack
VKAs: Vitamin K antagonists
